# Prevalence, risk factors and association with delivery outcome of curable sexually transmitted infections among pregnant women in Southern Ethiopia

**DOI:** 10.1371/journal.pone.0248958

**Published:** 2021-03-24

**Authors:** Mengistu Hailemariam Zenebe, Zeleke Mekonnen, Eskindir Loha, Elizaveta Padalko

**Affiliations:** 1 School of Medical Laboratory Sciences, Hawassa University college of Medicine and Health Sceinces, Hawassa, Ethiopia; 2 School of Medical Laboratory Sciences, Jimma University Institute of Health, Jimma University, Jimma, Ethiopia; 3 Department of Diagnostic Sciences, Ghent University, Ghent, Belgium; 4 Centre for International Health, University of Bergen, Bergen, Norway; 5 Chr. Michelsen Institute, Bergen, Norway; 6 Laboratory of Medical Microbiology, Ghent University Hospital, Ghent, Belgium; University of Pretoria, SOUTH AFRICA

## Abstract

**Introduction:**

Curable sexually transmitted infections (STIs) such as infection with *Chlamydia trachomatis* (*C*. *trachomatis*), *Neisseria gonorrhoeae* (*N*. *gonorrhoeae*), and *Trichomonas vaginalis* (*T*. *vaginalis*) can lead to adverse pregnancy and birth outcome. There are limited data on the prevalence and correlate of STI in Ethiopia, yet pregnant women are not screened for curable STI. Hence in this study, the prevalence of STIs and associated risk factors were assessed.

**Methodology:**

A cross- sectional study was conducted on consecutive women attending the delivery ward at the Hawassa comprehensive and specialized hospital. Vaginal swabs collected at the time of labor and delivery were tested for *C*. *trachomatis*, *N*. *gonorrhoeae* and *T*. *vaginalis* using GeneXpert. Study participants responded to a questionnaire about their previous and current obstetric history and socio-demographic characteristics. Possible independent factors for curable STIs were assessed by chi-square, bivariable, and multivariable, logistic regression.

**Results:**

Of the 350 vaginal swabs tested, 51 (14.6%, 95% CI: 10.9–18.3) were positive for one or more curable STIs. The prevalence of *C*. *trachomatis*, *N*. *gonorrhoeae* and *T*. *vaginalis* were 8.3%, 4.3%, and 3.1%, respectively. STIs was associated (p<0.005) with the delivery outcomes birth weight and gestational age. A 3-fold increase in odds of acquisition STIs was found in currently unmarried women (AOR, 3.5; 95% CI: 1.1–10.4; p = 0.028), in women <25 years (AOR, 2.7; 95% CI 1.1–6.6; p = 0.031). Women reporting presence of vaginal discharge (AOR, 7.7; 95% CI: 3.2–18.6; p < 0.001) and reporting pain during urination (AOR, 6.5; 95% CI: 2.6–16.2; p <0.001) found to associate with curable STIs.

**Conclusion:**

The higher magnitude of STIs found in this population, and the absence of symptoms in many illustrate the need for systematic follow-up during routine antenatal care primarily history taking and asking for signs and symptoms to provide early management and avoid long term sequelae.

## Introduction

Sexually transmitted infections (STIs) in pregnant women cause a significant global health burden related to various adverse health outcomes especially among women in developing countries [1]. Curable STIs consist of infections with *Chlamydia trachomatis* (*C*. *trachomatis*), *Neisseria gonorrhoeae* (*N*. *gonorrhoeae*), and *Trichomonas vaginalis* (*T*. *vaginalis*) [2]. Major complications of these infections include pelvic inflammatory disease, risk of ectopic pregnancy, spread to the foetus resulting in spontaneous abortion, preterm delivery, low birth weight, and stillbirth [1, 3]. Besides this, STIs have enormous social and economic consequences: marital conflict may occur when one of the partners develops STI or infertility [4].

Estimates of the prevalence and incidence of curable STIs remain high, with approximately over one million new infections each day [5, 6]. According to the WHO’s 2016 estimate, the prevalence of the four curable STIs among the reproductive age group of women is 3.8%, 0.9%, and 5.3% of *C*. *trachomatis*, *N*. *gonorrhoea*, and *T*. *vaginalis* respectively, while the African region shared the highest prevalence [6].

The Ethiopian health policy follows the WHO recommendation, advocating the syndromic management of curable STIs, encouraging pregnant women for regular surveillance and screening for syphilis and HIV. However, despite known high global incidence, curable STIs remain a neglected topic even in the area of research [7].

Since most STIs present asymptomatically, pregnant women, mainly in developing countries, rarely seek medical advice for this majority of them not diagnosed and treated [8]. For better approach, studies recommended screening pregnant women using the syndromic approach in the third trimester of pregnancy or before delivery as a solution to lessen bad outcome [9]

On the other hand, studies reported that aetiological diagnosis of STI remains difficult due to restricted access to laboratory diagnostics to guide appropriate treatment in developing countries. And even if facilities are available, test results for people with suspected STIs take days, making instant management of STI based on laboratory results impractical [10–12]. Because of these arguments, the WHO syndromic management has reached its limits and needs to be updated by integrating laboratory tests to address asymptomatic STIs. Previous studies also recommended the point-of-care tests (POCTs) that are accurate, rapid, simple and affordable are urgently desirable in resource‐constrained settings to support efficacious aetiological diagnosis and treatment [13].

Therefore in this study, the prevalence of curable STIs and their association with delivery outcomes were assessed using GeneXpert assay at Hawassa University Referral Hospital, Ethiopia.

## Methods

### Study setting and recruitment

Cross-sectional study was conducted among 350 consecutively enrolled pregnant women in the obstetrics ward at Hawassa University comprehensive and specialized hospital (HU-CSH), Ethiopia. From August to October 2020 consecutive pregnant women who came for delivery at the obstetric ward were recruited for the study after written informed consent. The HU-CSH is one of the teaching hospitals that serves as a referral centre for more than 5 million inhabitants in the Southern Region of Ethiopia. The hospital has 500 beds, accommodating around 2,504 pregnant women for antenatal care (ANC) visits and about 5,348 deliveries conducted annually.

### Sample size and technique

Sample size was estimated using a single population proportion formula with EPI-Info version 7. Considering the source population size greater than 50,000, the WHO prevalence estimate of *C*.*trachomatis* 3.8% [6], level of confidence 95%, margin of error 2%, and response rate 90%, sample size was calculated to be 349.

### Data collection and testing

A midwife at the obstetric ward provided general information about the study to pregnant women who came for delivery. Pregnant women agreeing to join in the study were interviewed using a structured questionnaire translated in Amharic, the language spoken by most people in the study area. The questionnaire was piloted on random mother at antenatal clinic to ensure the validity and feasibility of the questions as conducted in similar studies. Information related to socio-demographic characteristics (e.g., age, marital status, and educational level), obstetric history, and behavioral data were collected. The midwife-nurse took a vaginal swab specimen using Xpert CT/NG Vaginal/Endocervical specimen collection kits (Cepheid, Sunnyvale, California, USA). Swabs inserted in to the vaginal opening about 2 inches and gently turning around, ensuring rubbing the swab against the vaginal wall.

The collected samples were transported to the microbiology laboratory within 12 hours of collection. The trained microbiologists tested the specimen as per the manufacturer’s instruction (Xpert CT/NG and Xpert TV assays, Cepheid, Sunnyvale, California, USA). The Xpert assays have greater than 99% sensitivity and specificity for the organisms tested [14, 15]. The turnaround time for results was 60 minutes for *T*. *vaginalis* and 90 minutes for *C*. *trachomatis*/ *N*. *gonorrhoeae*.

### Ethical considerations

The ethics review committee of Hawassa University (CMHS/283/2012), Jimma University (IHRPGD/458/2020), National Health Research Ethics Review Committee (SRA/14.1/ 144483/2020) Ethiopia, and Ghent University (PA2019-038/BC-08458) Belgium, approved the study. All participants provided written, informed consent for study participation. Women who had curable STIs were linked to their physician and treated according to the national guideline [16]. Likewise, womens partner were referred for testing and treatment for STIs.

### Data analysis

Descriptive statistics were used to characterize the socio-demographic and obstetric and medical characteristics of the participants. We evaluated the prevalence of STI and factors associated using a logistic regression model after adjusted for a priori variables including mother’s age, educational level, marital statues and occupation. Finally, multivariable logistic regression was used to identify characteristics independently associated with having an STI and adjusting to other factors. Variables with a significant level of <0.2 were included in the final model. Statistical significance of variables in the final model was assessed at the 0.05 level. SPSS software version 20.0 (SPSS Inc. Chicago, IL, USA) was used for all analyses.

## Results

A total of 350 pregnant women were recruited and tested for curable STIs in this study. Participants’ mean age was 26.8, ranging from 17–41 years old and about 44% under 25 years. The majority (87.4%) of women were married, residing in an urban setting (79.1%), and Christian protestant (53%). Educationally more than half of the participants (63%) were above or at secondary level education (Table 1).

**Table 1:** Socio-demographic, obstetric history and behavioural characteristics of the 350 pregnant women and those with STI (n = 51).

Characteristics	Total (N = 350) n (%)	STI-Positive (n = 51) n (%)	p-value
**Age of mothers**			
<25	153 (43.7)	36 (23.5)	< 0.001
>25	197 (56.3)	15 (7.6)	
**Marital status**			
Unmarried	44(12.6)	15 (34.1)	0.020
Married	306 (87.4)	36 (11.8)	
**Residence**			
Urban	277(79.1)	41 (14.8)	0.812
Rural	73 (20.9)	10 (13.7)	
**Occupation**			
Self employed	82(23.4)	21 (25.6)	0.006
Government worker	107(30.6)	17 (15.9)	
Non-government worker	47(13.4)	3 (6.4)	
Student	46(13.1)	5 (10.9)	
Not employed	68(19.4)	5 (7.4)	
**Religion**			
Orthodox	76(21.7)	15 (19.7)	0.124
Protestant	187(53.4)	27 (14.4)	
Muslim	67(19.1)	7 (10.4)	
Catholic	11(3.1)	1 (9.1)	
Others	9(2.6)	1 (11.1)	
**level of Education**			
Primary and below	127(36.3)	27 (21.3)	0.008
Secondary and above	223 (63.7)	24 (10.8)	
**Gravidity**			
Primigravida	88(25.1)	11 (12.5)	0.525
Multigravida	262 (74.9)	40 (15.3)	
**ANC follow up**			
Yes	333(95.1)	50 (15)	0.319
No	17 (4.9)	1 (5.9)	
**History of preterm birth**			
Yes	22(6.3)	3 (13.6)	0.898
No	328 (93.7)	48 (14.6)	
**Previous history STIs**			
Yes	17(4.9)	4 (13.5)	0.290
No	333 (95.1)	47 (14.1)	
**Number of lifetime sexual partner**			
One	**295 (84.3)**	30 (10.2)	< 0.033
More than one	55(15.7)	21 (38.2)	

STIs, sexually transmitted infections; ANC, Antenatal care.

The majority of women (60.9%) gave birth through spontaneous vaginal delivery (SVD) and women who underwent caesarean section were 26.9%. Male to female ratio of newborn looked proportional, 47.4% male to 52.6% female. Three-fourths of participants had a previous pregnancy, and 95.1% had ANC follow up during their pregnancy. The majority of birth were alive (97.7%), and there were 6 (1.7%) stillbirth and 2 (0.6%) early neonatal death. About 79.7% of birth weight range between 2500–4000 gram and 15.5% of pregnant women gave preterm birth. Pregnant women STIs was associated (p<0.005) with delivery outcome of birth weight and gestational age. But has no with previous history of preterm birth and history of STIs (Tables 1 and 2).

**Table 2:** Curable sexually transmitted infection in relation to symptom and Birth outcome of pregnant women attending obstetric ward at Hawassa comprehensive and specialized hospital.

Characteristics	Total (N = 350) n (%)	STI-Positive (n = 51) n (%)	P-value
**Sex of newborn**			
Male	166 (47.4)	25 (15.1)	0.806
Female	184 (52.6)	26 (14.1)	
**Birth weight**			
<2500gram	33 (9.4)	13 (39.4)	< 0.001
**≥**2500 gram	317 (90.6)	38 (12.0)	
**Gestational age**			
Preterm	53(15.1)	18 (34)	< 0.001
Term	297 (84.9)	33 (11.1)	
**Birth outcome**			
Alive	342 (97.7)	49 (14.3)	
Stillbirth	6 (1.7)	2 (33.3)	0.213
Early neonatal death	2 (0.6)	0 (0)	
**Mode of delivery**			
SVD	213(60.9)	31 (14.6)	0.334
CS	94(26.9)	12 (12.8)	0.263
Episiotomy	31(8.9)	5 (16.1)	0.506
Forceps/vacuume extraction	12(3.4)	3 (25)	
**Vaginal discharge**			
Yes	59(16.9)	33 (55.9)	< 0.001
No	291 (83.1)	18 (6.2)	
**Pain during urination**			
Yes	57(16.3)	29 (50.9)	< 0.001
No	293 (83.7)	22 (7.5)	

SVD, Spontaneous vaginal delivery; CS, Caesarean section.

### Prevalence of curable STIs

STIs were detected in 14.6% (51/350) (95% CI: 10.9–18.3) of the 350 women. The prevalence was 8.3% (29/350) (95% CI: 5.1–11.1) for *C*. *trachomatis*, 4.3% (15/350) (95% CI: 2.3–6.9) for *N*. *gonorrhoeae*, and 3.1% (11/350) (95% CI: 1.4–4.9) for *T*. *vaginalis* (Table 3). Four pregnant women had a co-infection. From these; 3 women were positive for *N*. *gonorrhoeae* and *C*. *trachomatis* and one women for *C*. *trachomatis* and *T*. *vaginalis*.

**Table 3:** Pregnancy outcome and symptoms of sexually transmitted infections by laboratory confirmed STIs of study participant.

variables	*N*. *gonorrhoeae* n = 15	P-value	*C*. *trachomatis* n = 29	P-value	*T*. *vaginalis* n = 11	P-value
Age						
<25	11/153 (7.2)	0.018	21/153 (13.7)	0.001	8/153 (5.2)	0.064
>25	4/197 (2.0)		8/197 (4.1)		3/197 (1.5)	
Gestational age						
Term	9/297 (3.0)	0.015	21/297 (7.1)	0.060	6/297 (2.0)	0.015
Preterm	6/53 (11.3)		8/53 (8.3)		5/53 (9.4)	
Birth weight						
<2.5 Kg	6/33 (18.2)	0.001	6/33 (18.2)	0.043	4/33 (12.1)	0.014
>2.5 Kg	9/317 (2.8)		23/317 (7.3)		7/317 (2.2)	
Vaginal discharge						
Yes	9/59 (15.3)	<0.001	19/59 (32.2)	<0.001	8/59 (13.6)	<0.001
No	6/291 (2.1)		10/291 (3.4)		3/291 (1.0)	
Pain during urination						
Yes	5/57 (8.8)	0.078	20/57 (35.1)	<0.001	6/57 (10.5)	0.003
No	10/293 (3.4)		9/293 (3.1)		5/293 (1.7)	
Prevalence (%)	15/350 (4.3)		29/350 (8.3)		11/350 (3.1)	

Pregnant women’s age, vaginal discharge, gestational age and birthweight had significant association (p<0.05) with *N*. *gonorrhoeae* positivity. Likewise, symptom of STIs (vaginal discharge and pain during urination) and birthweight had association with *C*. *trachomatis* and *T*. *vaginalis* positivity (Table 3).

### STIs and associated factors

In a bivariable analysis after adjusted for a priori variables, STIs were more common among women who were currently unmarried, less than 25 years women, who give a preterm and leaser birth weight baby, women with lower educational status, women with symptom of vaginal discharge and pain during urination, and who had more than one sexual partner (Table 4).

**Table 4:** Bivariable and multivariable analysis of socio-demographic characteristics of pregnant women with curable sexually transmitted infections attending obstetric ward at Hawassa comprehensive and specialized hospital.

Characteristics	Curable STI Number (%)	Bivariate analysis		Multivariate analysis	
		COR	P-value	AOR	P-value
**Age**					
<25	36(23.5)	3.7(1.9–7.1)	< 0.001	2.7(1.1–6.6)	0.031
>25	15 (7.6)	1		1	
**Marital statues**					
Married	36(11.8)	1		1	
Unmarried	15(34.1)	3.9 (1.9–7.9)	0.020	3.4(1.1–10.4)	0.028
**Occupation**					
Self employed	21(41.2)	4.3(1.5–12.2)	0.006	7.6(1.7–34.2)	0.009
Government worker	17(15.9)	2.3(0.8–6.7)	0.105	3.7(0.9–15.8)	0.078
Non-government worker	3(6.4)	0.9(0.2–3.8)	0.841	2.3(0.4–15.4)	0.391
Student	5(10.9)	1.5 (0.4–5.6)	0.517	1.3(0.2–7.5)	0.795
Not employed	5 (7.4)	1		1	
**Education**					
Primary and below	27(21.3)	2.3(1.2–4.4)	0.017	2.3(0.9–5.4)	0.067
Secondary and above	223(63.7)	1		1	
**Gestational age**					
Term	33 (11.1)	1		1	*
Preterm	18(34)	3.7 (1.7–7.9)	0.001	2.4(0.9–6.8)	0.088
**Birth weight**					
<2500gram	13 (39.4)	6.8(2.7–16.9)	<0.001	5.9(1.8–19.1)	0.003
**≥**2500 gram	38 (12.0)	1		1	*
**Vaginal discharge**					
Yes	33(55.9)	14.4(6.8–30.8)	< 0.001	7.7(3.2–18.6)	< 0.001
No	18(6.2)	1		1	*
**Pain during urination**					
Yes	29(50.9)	11.4(5.3–24.7)	< 0.001	6.5(2.6–16.2)	< 0.001
No	22(7.5)	1		1	*
**Number of lifetime sexual partners**					
One	30(10.2)	1		1	*
More than one	21(38.2)	5.9 (2.7–12.8)	< 0.001	2.9(1.0–8.3)	0.048

* Model adjusted for maternal age, marital status, occupation and educational level.

STIs, sexually transmitted infections.

Multivariable logistic regression analysis indicates, younger women (<25 years) were at higher risk of having curable STI compared to elders (AOR, 2.7; 95% CI 1.1–6.6; p = 0.031). The other independent predictors associated with curable STIs were unmarried women (AOR, 3.4; 95% CI: 1.1–10.4; p = 0.028) compared to married women, self-employed women compared to un-employed (AOR, 7.6; 95%CI: 1.7–34.7; p = 0.009), underweight (AOR, 5.9; 95% CI: 1.8–19.1; p < 0.003) compared to normal birth weight, women reporting presence of vaginal discharge (AOR, 8.3; 95% CI: 3.4–20.5; p < 0.001) compared to no vaginal discharge (Table 4).

Women reporting pain during urination were six-fold infected by at least one curable STI than those with no pain (AOR, 6.5; 95% CI: 2.6–16.2; p <0.001) (Table 4). Having more than one sexual partner and an educational level of only junior or primary were identified as potential predictors of curable STIs in the bivariable analysis but not multivariable analysis.

## Discussion

Among pregnant women who attended delivery at the Hawassa comprehensive and specialized hospital, 14.6% tested positive at least for one of the three curable STIs: *C*. *trachomatis* 8.3%, *N*. *gonorrhoeae* 8.3%, and *T*. *vaginalis* 3.1%. A statistically significant association was observed between STIs and variables like age (<25 years), being unmarried, having lower or primary education, having vaginal discharge, and pain during urination.

The prevalence of *C*. *trachomatis* in the present study is within the range and somehow comparable (0–31.1%, with a pooled prevalence of 6.9%) to a systematic review in sub-Saharan Africa compiled by Kristina Adachi et al.; [17]. Another systematic review of low and middle-income countries revealed a mean prevalence of 4.2% of *C*. *trachomatis* in east Africa, which is lower than our results [7]. Results comparable to our finding for *C*. *trachomatis* were reported in a systematic review with a pooled prevalence of 7.8% in Sub-Saharan Africa among reproductive-age women [18]; 8% in Botswana [19], and 9.8% in central Ethiopia [20]. On the other hand, our findings were substantially lower compared to previous findings: 18.9% in the same area (Hawassa) [21] using rapid antigen test kit, 14.9% in Kenya [22], 20% in South Africa [23] and 26.5% among HIV positive pregnant women in South Africa [24]. In this study women’s age, vaginal discharge, and birthweight had significant association with *C*. *trachomatis* positivity.

The rate of *T*. *vaginalis* in this study was lower than findings from previous studies; 4.98% in Jimma, Ethiopia [25] using culture system, 5.3% in central Ethiopia [20] using rapid test kits, 7.4% in Kenya [22] using GeneXpert, 9.1% in Nigeria [26] using microscopic method, 15% in South Africa [23] and 16.7% among HIV positive pregnant women in South Africa [24]. This might be due difference in laboratory assays used besides socio-demographic variation in of study population. Our study presented significant differences in gestational age, birth weight and symptom of STIs (vaginal discharge and pain during urination) among pregnant women with and without *T*. *vaginalis*.

Similarly, the prevalence of *N*. *gonorrhoea* in the present study was slightly lower than previously published results in the same study area, 5.1% [27] done using culture techniques but comparable with the study (4.3%) in central Ethiopia [20] using rapid test kits. However, our result was higher than the report from another study’s report in Hawassa (0.31%) [21] using rapid antigen test kit. Again the possible reasons might be due to different laboratory procedures applied. Also our study showed that maternal *N*. *gonorrhoea* positivity had significant association with gestational age, birthweight and mother’s age groups. Previous studies also has reported that birthweight and gestational age at birth has significant association with maternal *N*. *gonorrhoea infection* [1].

Age was found to be a significantly associated factor with STIs. Young age (<25 years) had a three-fold increased risk for curable STIs, which is in line with previous similar findings [21, 27]. It has been speculated that more sexually active behaviour is seen among youngsters compared to their elders. Other studies also reported a younger maternal age as a risk factor for STIs [28]. Hence as indicated by Centre for disease control and prevention (CDC) of America, at the first prenatal visit screening all pregnant women (mainly younger women <25 years) for curable STIs is mandatory. The fact that most curable STIs are silent or asymptomatic if young women get infected, they might not distinguish the sign of pregnancy from STI sourced vaginal discharge and pain during urination due to lack of experience [29]

Pregnant women with curable STIs had a 4-fold increase odds of giving preterm birth. This finding was in agreement with studies, which revealed STIs have been implicated in adverse pregnancy outcomes including preterm delivery and low birth weight [30, 31]. Likewise, women with curable STIs were at higher odds of giving low birth weight even though were not included in the final models. Both preterm birth and low birth weight are leading factors of neonate morbidity and mortality, particularly in developing countries where neonatal intensive care facilities are not often available [12, 30, 32].

In this study number of sexual partners of women found to be associated with STIs. Also symptoms such as vaginal discharge and pain during urination were significantly associated with STI. This association has similarities with study reports from Ethiopia [25, 33]. Although maternal healthcare service utilization is an important predictor of favourable maternal and child health outcomes, in Ethiopia only a few health centres offer STI diagnosis. Therefore the Ethiopian ANC follow-up policy should focus on addressing the possible strategies in STI prevention among pregnant women during their ANC visit to visualised healthy alive birth. In addition to compelling evidence that demands the development and validation of point-of-care tests for STIs [9].

Compared to the pooled stillbirth rate of Ethiopia (3.7%) and early neonatal death of (2.9%), compiled through systematic review, our result shows some reduction, 6 (1.7%) stillbirth and 2 (0.6%) early neonatal death [34]. On the other hand, although 15.5% of pregnant women gave pre-term birth, it lacks any association with women STIs. Similarly, association was not observed with the mode of delivery, ANC follow-up and gravidity of women, however, literature showed the association of STIs with these predictors [33, 35].

As to the World Health Organization, pregnant women should visit antenatal care at least four times during pregnancy aimed to risk identifications, prevention and management of pregnancy related diseases, and health education and health promotion [36]. However, as syndromic approach offers treatment for a group of diseases the lack of standard of antenatal care and overlooking curable STIs in Ethiopia has significant consequence for developing fetus beyond. In this study even though the symptom of STIs has an association with STIs positivity, STIs were detected in symptomless women too. This implicates that syndromic management can miss and left untreated some of the women that rises adverse consequences. Also it has been reported in previous study in central Ethiopia that the higher rate of maternal STIs and its impact on the unborn child demonstrate the need for screening and treatment programmes in order to prevent sequel [20]. Therefore, such type of findings demand serious attention in order strengthening the existing ANC services through integration of diagnostic for curable STIs rather than exclusively depend on the syndromic management.

As the study was a facility-based, it could not show the overall picture of STIs in the source population where ANC follow-up is not an optimum level. Besides, we could not verify the time when the women got infected as it was a cross-sectional study. Subsequent effects on babies delivered from those STIs positive women were not studied. Meanwhile, the wider confidence interval of the effect measures for some variables implies the need to consider a larger sample size in future studies of the same.

## Conclusion

The high prevalence of STIs in the study area continue to have an impact on pregnancy outcome. The absence of symptoms in many illustrates the need for systematic follow-up during routine antenatal care primarily history taking and asking for signs and symptoms of an STI. In the meantime, the syndromic approach needs to be updated by integrating antenatal screening services for curable STIs through the provision of affordable, rapid, point-of-care screening tests in resource-constrained antenatal care settings.

## Supporting information

S1 File PDF English version questioner. (DOCX)

S2 File PDF Amharic version questioner. (DOCX)

S1 Dataset SPSS data. (SAV)
