# Supplementary material for: Prevalence, risk factors and association with delivery outcome of curable sexually transmitted infections among pregnant women in Southern Ethiopia
Source: PLoS One. 2021 Mar 24;16(3):e0248958. doi: 10.1371/journal.pone.0248958 (PMC7990168; doi:10.1371/journal.pone.0248958)
Supplement: S1 File — (DOCX) [file pone.0248958.s001.docx]

Lab code_______________ . Patient ID. ________________ Date ________

|  | **Inquiries** | | **Response**  **(circle clients response)** |
| --- | --- | --- | --- |
|  | |  |  |
|  | Birth weight of new born | |  |
|  | Birth status | | Term/post term  Preterm |
|  | Sex of new born | | Male  Female |
|  | |  |  |
|  | Age of mother | |  |
|  | Place of residence | | Rural  Urban |
|  | Your Marital status | | Married  Currently unmarried |
|  | Occupational status? | | Self-employed  Government employed  Private employed  Not-employed |
|  | Annual income (ETH birr) | | Low (<15,000)  Medium( 15001- 48000)  High (>48000) |
|  | Are you health care worker? | | Yes  No |
|  | Are you working at day care? | | Yes  No |
|  | Your religion | | Orthodox  Protestant  Muslim  Catholic  Other |
|  | Your Educational level | | Primary school or below  Secondary school or above |
|  | Do you have children? | | Yes  No |
|  | Do you have children attending day care? | | Yes  No |
|  | Gravidity | | Primigravida  Multigravida |
|  | Do you have any check-up during current pregnancy? | | Yes  No |
|  | How many time you have checked? | |  |
|  | Do you have any history of premature births? | | Yes  No |
|  | Have you ever had a child who had an infection at birth? | | Yes (if yes to Q 20)  No |
|  | What was the evolution? | | Good  Death  Disability |
|  | Have you ever had a sexually transmitted infection? | | Once  More than once  Never |
|  | How many total number of sex partners (life time) have you had | | One  More than 1 |
|  | Do you have any history of abortion? | | Yes  No |
|  | Do you have any history of stillbirth? | | Yes  No |
|  | |  |  |
|  | Do you have contact with children attending at day care? | | Yes  No |
|  | Do you share the same cup with your child? | | Yes  No |
|  | Do you share a food with your child? | | Yes  No |
|  | Do you share eating utensils (fork or spoon) with your child? | | Yes  No |
|  | Do you share a toothbrush with a young child? | | Yes  No |
|  | Do you eat raw vegetables, fruits and/or salads? | | Not once  Sometimes  Frequently |
|  | Do you wash vegetables, fruits and/or salads before you eat them raw? | | Not once  Sometimes  Frequently |
|  | Do you eat raw or under cooked meat? | | Yes  No |
|  | Do you drink raw or under boiled milk? | | Yes  No |
|  | What is your usual source of water for daily use? | | Open source wells  Tap water  Other, please specify: |
|  | Do you have domestic cats in your home? | | Yes  No |
|  | Do you have contact with cats in your home? | | Yes  No |
|  | Have you heard about congenital transmitted infection? | | Yes  No |
|  | Where did you obtain this knowledge? (checking more than one checkbox is possible | | Doctor  Internet  Peers  Other, please Specify |

**Thank You for participation**

Signature of interviewer: _________________ Date: _____/_____/_________

Signature of study Coordinator: _____________________ Date: _____/_____/_________
