# Supplementary material for: Prevalence, risk factors and association with delivery outcome of curable sexually transmitted infections among pregnant women in Southern Ethiopia
Source: PLoS One. 2021 Mar 24;16(3):e0248958. doi: 10.1371/journal.pone.0248958 (PMC7990168; doi:10.1371/journal.pone.0248958)
Supplement: S2 File — (DOCX) [file pone.0248958.s002.docx]

በላብራቶሪ የተሰጠው መለያ ቁጥር____________ የበሽተኞ መለያ ቁጥር____________ ቀን__________

|  | **ጥያቄዎች** | **የደንበኞችን ምላሽ ምልክት ያድርጉበት** |
| --- | --- | --- |
|  | የተወለደው ህፃን ክብደት | ________________ |
|  | የተወለደው ህፃን ሁኔታ | በጊዜው የተወለደ  ያለጊዜው የተወለደ |
|  | የተወለደው ህፃን | ወንድ  ሴት |
|  | የእናት የትውልድ ቀን | _____________ |
|  | የመኖሪያ ቦታ | ገጠር  ከተማ |
|  | የጋብቻ ሁኔታ | ያላገባ  ያገቡ |
|  | የሰራ ሑኒታ | በግል ተዳዳሪ  መንግሥት ተቀጣሪ  በግል ተቀጣሪ  ስራ የሊላት |
|  | የሶሺዮ-ኢኮኖሚያዊ ሁኔታ | ዝቅተኛ  መካከለኛ  ከፍተኛ |
|  | የጤና እንክብካቤ ሠራተኛ ነዎት? | አዎ  አይ |
|  | በልጆች መዋእለ ሕፃናት (ማቆያ) ውስጥ እየሰሩ ነው? | አዎ  አይ |
|  | ሃይማኖት | ኦርቶዶክስ  ፕሮቴስታንት  ሙስሊም  ካቶሊክ  ሌላ |
|  | የእርስዎ የትምህርት ደረጃ | መደበኛ ትምህርት ያልተከታተለ/ የመጀመሪያ ደረጃ ትምህርት ቤት  ሁለተኛ ደረጃ ትምህርት ቤት/የዩኒቨርሲቲ ደረጃ |
|  | ልጆች አለዎት? | አዎ  አይ |
|  | መዋእለ ሕፃናት (ማቆያ) የሚማሩ ልጆች አሉዎት? | አዎ  አይ |
|  | የቀደሙ እርግዝናዎች? | አዎ  አይ |
|  | በአሁኑ እርግዝና ወቅት የሐኪም ክትትል አለዎት? | አዎ (ለ Q16 አዎከሆነ)  አይ |
|  | ስንት ጊዜ ታይቷዎል? |  |
|  | ያለጊዜው የተወለዱ ልጅ አለዎት? | አዎ  አይ |
|  | ሲወለድ የጠና ችግር የገጠመው ልጅ ነበረዎት? | አዎ (አዎለ Q 21 ከሆነአዎ)  አይ |
|  | ሲወለድ ኢንፌክሽኑ የተከሰተ ልጅ አለዎት? | አዎ (አዎን ከሆነ 21 )  አይ |
|  | ውጤቱስ ምን ሆነ? | ጤናማ  ሞት  የአካል ጉዳት |
|  | እስካሁን ምን ያህል የወሲብ ጓደኛ አለዎት? | አንድ ብቻ  ከአንድ በላይ |
|  | በግብረስጋ ግንኙነት የሚተላለፍ በሽታ አጋጥሞ ያውቃል? | አንዴ  ከአንድ ጊዜ በላይ  በጭራሽ |
|  | ፅንስ ማስወረድ አጋጥሞ ያውቃል? | አዎ  የለም |
|  | የፅንስ መጨንገፍ አጋጥሞ ያውቃል? | አዎ  የለም |
|  | በሕፃናት ማቆያ የሚቆይ ልጆት ጋር ግንኙነት አለዎት? | አዎ  አይ |
|  | በተመሳሳይ ኩባያ ለመጠጣት ከልጅዎ ጋር ይጋራሉ? | አዎ  አይ |
|  | ከልጅዎጋር ምግብ ይጋራሉ? | አዎ  አይ |
|  | የመመገቢያ ቁሳቁሶችን (ሹካ ወይም ማንኪያ) ከልጅዎ ጋር ይጋራሉ? | አዎ  አይ |
|  | የጥርስ ብሩሽ ከትንሽ ልጅ ጋር ይጋራሉ? | አዎ  አይ |
|  | ጥሬ አትክልቶችን፣ፍራፍሬዎችን እና / ወይም ሰላጣዎችን ይበላሉ? | በጭራሽ  አንዳንድ ጊዜ  በተደጋጋሚ |
|  | አትክልቶችን፣ፍራፍሬዎችን እና / ወይም ሰላጣዎችን ጥሬ ከመመገባቸው በፊት ያጥባሉ? | በጭራሽ  አንዳንድ ጊዜ  በተደጋጋሚ |
|  | ጥሬ ወይም ያልበሰለ ስጋ ትመገባላችሁ? | አዎ  አይ |
|  | ያልፈላ ወተት ይጠጣሉ? | አዎ  አይ |
|  | ለዕለታዊ አገልግሎት የተለመደው የውሃ ምንጭዎ ምንድነው? | የጉድጓድ ውሃ  የቦንቦ ውሃ  ሌላ፣እባክዎን ይጥቀሱ |
|  | በቤትዎ ውስጥ ድመቶች አሉዎት? | አዎ  አይ |
|  | በቤትዎ ውስጥ ካሉ ድመቶች ጋር ንክኪ አለዎት? | አዎ  አይ |
|  | ከነፍሰጡር ሴቶች ወደ ልጆች ስለሚተላለፍ ኢንፌክሽን ሰምተው ያውቃሉ? | አዎ  አይ |
|  | ከነፍሰጡር ሴቶች ወደ ልጆች ስለሚተላለፍ ኢንፌክሽን መረጃ ያገኙት ከየት ነው? | ከዶክተር  በዜና  ከጓደኞች  ሌላ፣እባክዎንይግለጹ |

ስለተሳተፉእናመሰግናለን::

የቃለ-መጠይቅ ጠያቂ ፊርማ_________________ ቀን _____ / _____ / _________

የጥናት አስተባባሪ ፊርማ_____________________ ቀን _____ / _____ / _________
